# Preventive and Therapeutic Euphol Treatment Attenuates Experimental Colitis in Mice

**DOI:** 10.1371/journal.pone.0027122

**Published:** 2011-11-02

**Authors:** Rafael C. Dutra, Rafaela F. Claudino, Allisson F. Bento, Rodrigo Marcon, Éder C. Schmidt, Zenilda L. Bouzon, Luiz F. Pianowski, João B. Calixto

**Affiliations:** 1 Department of Pharmacology, Centre of Biological Sciences, Universidade Federal de Santa Catarina, Florianópolis, Brazil; 2 Department of Cell Biology, Embryology and Genetics, Central Laboratory of Electron Microscopy, Universidade Federal de Santa Catarina, Florianópolis, Brazil; 3 Pianowski and Pianowski Ltda., São Paulo, Brazil; Université Paris Descartes, France

## Abstract

**Background:**

The tetracyclic triterpene euphol is the main constituent found in the sap of *Euphorbia tirucalli*. This plant is widely known in Brazilian traditional medicine for its use in the treatment of several kinds of cancer, including leukaemia, prostate and breast cancers. Here, we investigated the effect of euphol on experimental models of colitis and the underlying mechanisms involved in its action.

**Methodology/Principal Findings:**

Colitis was induced in mice either with dextran sulfate sodium (DSS) or with 2,4,6-trinitrobenzene sulfonic acid (TNBS), and the effect of euphol (3, 10 and 30 mg/kg) on colonic injury was assessed. Pro-inflammatory mediators and cytokines were measured by immunohistochemistry, enzyme-Linked immunoabsorbent assay (ELISA), real time-polymerase chain reaction (RT-PCR) and flow cytometry. Preventive and therapeutic oral administration of euphol attenuated both DSS- and TNBS-induced acute colitis as observed by a significant reduction of the disease activity index (DAI), histological/microscopic damage score and myeloperoxidase (MPO) activity in colonic tissue. Likewise, euphol treatment also inhibited colon tissue levels and expression of IL-1β, CXCL1/KC, MCP-1, MIP-2, TNF-α and IL-6, while reducing NOS2, VEGF and Ki67 expression in colonic tissue. This action seems to be likely associated with inhibition of activation of nuclear factor-κB (NF-κB). In addition, euphol decreased LPS-induced MCP-1, TNF-α, IL-6 and IFN-γ, but increased IL-10 secretion from bone marrow-derived macrophages *in vitro*. Of note, euphol, at the same schedule of treatment, markedly inhibited both selectin (P- and E-selectin) and integrin (ICAM-1, VCAM-1 and LFA-1) expression in colonic tissue.

**Conclusions/Significance:**

Together, these results clearly demonstrated that orally-administered euphol, both preventive or therapeutic treatment were effective in reducing the severity of colitis in two models of chemically-induced mouse colitis and suggest this plant-derived compound might be a potential molecule in the management of inflammatory bowel diseases.

## Introduction

Inflammatory bowel diseases (IBD) are a group of chronic disease that affect the gastrointestinal tract and have been mainly subdivided as ulcerative colitis (UC) and Crohn’s disease (CD). Although its etiopathogenesis has not been definitively elucidated, it is currently considered an abnormal inflammatory response to intestinal microbial flora. However, there is still not consensus whether or not IBD should be considered as an autoimmunity, since the loss of tolerance is towards gut microbiota and the autoantigen in the human body is still missing [1]. IBD is now considered as a result of continuous microbial antigenic stimulation of pathogenic immune responses as a consequence of host genetic defects in mucosal barrier function, innate bacterial killing or immunoregulation [2].

CD is characterized by T_H_ type-1 response, marked by up-regulation of interferon-γ (IFN- γ), a key cytokine, which mainly driven T cells. On the other hand, UC appears to exhibit an contribution of T_H_2 responses (characterized by secretion of interleukin (IL)-4 and IL-13), which driven NK-T cells [3], although this difference in immune response is not yet well established. Interestingly, it was observed that in both CD and UC some pro-inflammatory mediators such as tumour necrosis factor-α (TNF-α), IL-6 and IL-1β are released in inflamed mucosa.

It is now well recognized that inflammation of the intestinal mucosa is characterized by chronic inflammatory cell infiltration composed mainly of neutrophils and macrophages, an effect that is accompanied by production of pro-inflammatory cytokines, like IL-1β, IL-6, TNF-α and IFN-γ [4]. In addition, the recruitment of these cells from the vasculature to sites of inflammation is dependent on a multistep cascade of adhesive interactions, which are mediated by adhesion molecules and chemoattractants, such as, integrins/selectins and chemokines, respectively [5], [6].

Over the past decade, a substantial amount of evidence has been generated to support a crucial role of leukocytes in the pathogenesis of colitis [7], [8]. The process of leukocyte extravasations is a critical step in the inflammatory response and is dependent on three different families {Barreiro, #603}. (1) Selectins belong to a family of three carbohydrate-recognizing molecules: E-selectin, which is mainly expressed on activated endothelium; P-selectin, which is expressed on platelets and the endothelium; and L-selectin, which is constitutively expressed on leukocytes [10]. Several studies using antibody blockade of selectins have demonstrated the relevant role of selectins in leukocyte rolling [11], [12]. (2) Integrins comprise a family of 24 heterodimeric receptors, each of which is composed of an α- and a β-subunit. These molecules dynamically alter their adhesive properties through conformational changes (affinity) as well as through spatial redistribution on the cell surface and are fundamental molecules in cell migration [13], [14]. Furthermore, integrins can recognize multiple ligands including proteins of the extracellular matrix, cell surface glycoprotein as well as complement factors and soluble components of the hemostatic and fibrinolytic cascade. (3) The major integrin ligands involved in leukocyte adhesion belong to the immunoglobulin superfamily [15] and include intercellular cell adhesion molecules (ICAM) 1–5, vascular cell adhesion molecule-1 (VCAM-1) and junctional adhesion molecules (JAMs), which are expressed on endothelial and other types of cells [16]. Thus, blocking adhesion molecules constitutes a potential and useful target for controlling leukocyte influx into the site of inflammation and consequently, leads to the development of new anti-inflammatory drugs.

**Figure 1:**
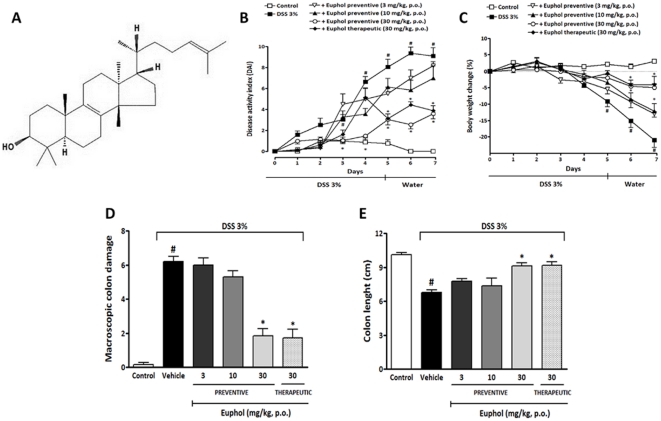
Euphol ameliorates DSS-induced acute colitis. (A), Chemical structure of euphol. Mice received DSS for 5 days and drinking water for the next 2 days. Animals were orally treated by gavage with 3, 10, or 30 mg/kg of euphol twice a day from day 0 to day 7 (preventive treatment) or with 30 mg/kg from day 3 to day 7 (therapeutic treatment). Preventive or therapeutic oral treatment with euphol improved the disease activity index (DAI) score (B), reduced body weight loss (C) and colon macroscopic damage (D), and enhanced colon length (E) when compared with mice from the DSS group. Data are reported as means ± S.E.M. of 8 to 10 mice per group and is representative of three independent experiments. ^#^P<0.05 vs. control healthy group; *P<0.05 vs. DSS-treated group.

The tetracyclic triterpene euphol (Fig.1A) is the main constituent found in the sap of *Euphorbia tirucalli*, a plant belonging to the family Euphorbiaceae, and known in Brazilian traditional medicine as aveloz, árvore-do-lápis, cega-olho or espinho-italiano. In the northeast region of Brazil, the latex of *E. tirucalli* is used as a folk therapy against syphilis, laxative agent, to control intestinal parasites, to treat asthma, cough, earache, rheumatism, cancer, chancre, epithelioma, sarcoma and skin tumors [17]. The bark/latex of *E. tirucalli* presents pharmacological activities as molluscicide, antiherpetic and anti-mutagenic [17]. It also shows co-carcinogenic and anti-carcinogenic activities [18]. In addition, the sap of *E. tirucalli* showed relevant potential larvicide against *Aedes aegypti* and *Culex quinquefasciatus*, the most common dengue vector and lymphatic filariasis vector, respectively [19] and demonstrated higher piscicidal activity as compared with other synthetic pesticides, organophosphates and pyrethroids for the fish *Heteropneustes fossilis* [20]. Furthermore, a very recent study demonstrated that biopolymeric fraction (BET) from plant *E. tirucalli* showed dose dependent anti-arthritic activity and demonstrated *in vivo* suppression of CD4^+^ and CD8^+^ T cells associated with inhibition of intracellular IL-2 and IFN-γ [21]. Our group has previously shown that euphol administered orally elicited pronounced and long-lasting analgesia when assessed in several rodent behavior models of inflammatory and neuropathic persistent pain and these actions were likely to be associated with the inhibition of TNF-α and IL-1β levels, as well as inhibition of transcription factors, such as nuclear factor-κB (NF-κB) and cyclic AMP response element-binding protein (CREB), both in the spinal cord and dorsal root ganglia (unpublished data).

Despite great progress observed over the previous decades in understanding the cellular and molecular mechanisms involved in IBD, few effective and safe drugs have emerged to treat acute and chronic inflammatory bowel states. Therefore, new effective treatment for IBD is urgently needed. Hence, in the present study, we investigated the preventive and therapeutic potential effects of euphol in dextran sulfate sodium (DSS)- and 2,4,6-trinitrobenzene sulfonic acid (TNBS)-induced colonic inflammation in mice. Herein, we report that the tetracyclic triterpene euphol can effectively ameliorate DSS- and TNBS-induced colitis by inhibiting pro-inflammatory mediators such as cytokines/chemokines in the colonic tissue and in primary cultures of macrophages *in vitro*. Our data also indicated that the mechanisms underlying the anti-inflammatory activity of euphol was likely to be related with its ability to inhibit selectin and integrin expression in the endothelium, associated with the blocking of nitric oxide synthase-2 (NOS2), vascular endothelial growth factor (VEGF), and Ki67 expression in colonic tissue by modulating the nuclear transcription factor-κB (NF-κB).

## Results

### Euphol treatment attenuates the severity of DSS-induced acute colitis

In mice with DSS-induced acute colitis, which resembles the acute phase of human ulcerative colitis, we observed hemorrhage in the colonic lumen, body weight loss and marked diarrhea with bloody stools, which ultimately resulted in a sharp increase of the disease activity index (DAI) from day 3 onwards, compared with control healthy (non-colitic) mice (Fig. 1). Euphol was administered orally at three different doses (3, 10 and 30 mg/kg) to detect potential dose-dependent effects. Preventive oral treatment with 30 mg/kg of euphol but not with 3 or 10 mg/kg significantly reduced the DAI from day 3 onwards (Fig. 1B). DSS administration was associated with significant body weight loss on days 5, 6 and 7. Mice treated with euphol (30 mg/kg) were protected from marked body weight loss and recovered a healthy appearance that was similar to that of control healthy mice (non-colitic) (Fig. 1C). DSS administration resulted in colon inflammation associated with hyperemia, ulceration and bowel wall thickening, leading to an increase in macroscopic colon damage and decrease in colon length (Fig. 1 D,E). Oral euphol (30 mg/kg) treatment reduced macroscopic damage (Fig. 1D) and significantly prevented colon length reduction (Fig. 1E) on the 7^th^ day after colitis induction. In contrast, treatment with euphol at doses of 3 or 10 mg/kg, p.o., was not able to reduce macroscopic damage, prevent colon length reduction and recover the loss in body weight. Based on these results, the dose of 30 mg/kg of euphol was used in subsequent experiments to investigate some of the mechanisms underlying its anti-inflammatory effects.

Next, we determined whether euphol also had a therapeutic effect on DSS-induced acute colitis by administering it after colitis symptoms were seen on day 3, since although preventive treatments are important, treatments that have efficacy after colitis symptoms have been established are more clinically relevant. The results for the disease activity index (Fig. 1B), body weight loss (Fig. 1C), macroscopic damage (Fig. 1D) and colon length (Fig. 1E) showed that the therapeutic effect of euphol (30 mg/kg, p.o.) was as effective as its preventive effect in alleviating the severity of DSS-induced acute colitis.

Euphol treatment inhibited polymorphonuclear leukocyte influx and reduced colon damage

**Figure 2:**
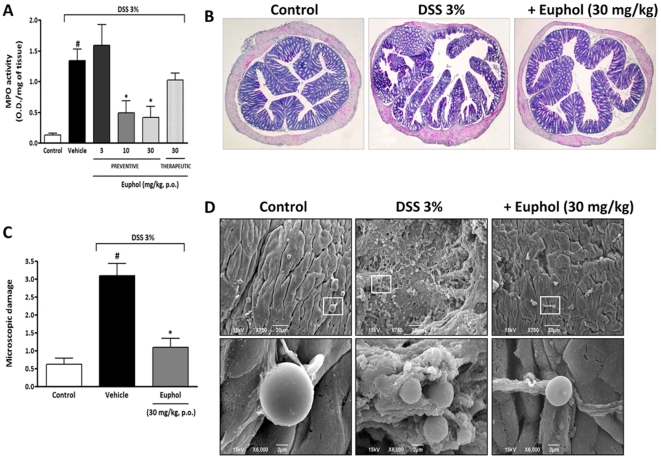
Treatment with euphol reduces cell influx and microscopic colon damage after DSS-induced acute colitis. At 7 days after euphol oral treatment, colon tissues were processed for histological evaluation, measurement of myeloperoxidase (MPO) activity and scanning electron microscopy. Preventive (3, 10, and 30 mg/kg, p.o.) or therapeutic (30 mg/kg, p.o.) treatment with euphol reduced MPO (A) activity. (B) Representative histological sections of colon from control healthy mice (non colitic), DSS-treated and euphol-treated mice (30 mg/kg, p.o.) were examined microscopically after H&E staining with original magnification x20. The images are representative of at least four mice per group. (C) Preventive treatment with euphol (30 mg/kg, p.o.) decreased the microscopic damage score in mouse colon. (D) Scanning electron microscopy photographs of the colon of the colon surfaces of control healthy mice, DSS-treated group, and DSS plus euphol (30 mg/kg, p.o.) treated mice after 7 days following DSS administration. Original magnification: x750 and x6,000, respectively. Each column represents the mean ± S.E.M. of 8 to 10 mice per group and is representative of two independent experiments. ^#^P<0.05 vs. control healthy group (non colitic); *P<0.05 vs. DSS-treated group.

Several studies have suggested that tissue damage and inflammatory signals in experimental colitis are mainly mediated by polymorphonuclear leukocytes (PMN), mainly neutrophils [6], [8]. We therefore assessed whether the effect of euphol treatment in DSS-mediated colitis was associated with alterations in the composition of the neutrophil population in the intestinal mucosa. Seven days after the initiation of DSS treatment, mucosal neutrophils infiltration into the colon was indirectly assessed by measuring MPO activity. Colonic samples taken from untreated (DSS) mice displayed significantly increased MPO levels relative to control healthy mice (non-colitic) (Fig. 2A). Notably, preventive oral treatment with euphol at 10 and 30 mg/kg, but not 3 mg/kg, significantly reduced DSS-induced increase in colonic MPO levels (Fig. 2A). Of note, therapeutic treatment with euphol (30 mg/kg, p.o.) also inhibited MPO levels (Fig. 2A). To further investigate the effect of euphol treatment in the architecture and integrity of colonic structure and confirm results from MPO analysis, colons were processed for histological observation. Mice with colitis induced by DSS exhibited disruption of the epithelial barrier, a marked decrease in the number of crypts, and marked infiltration of inflammatory cells, predominantly neutrophils, into the mucosa and sub mucosa of the colon, corroborating the MPO assay (Fig. 2B). The histological evaluation of colons from euphol-treated (30 mg/kg, p.o.) mice revealed a pronounced reduction in the inflammatory response with moderate loss of epithelial cells and minimal inflammatory infiltration into the colonic tissue, resulting in a decreased microscopic damage score, compared with colons from DSS mice (untreated) (Fig. 2 B,C).

### Euphol prevents epithelial surface lesion in colonic tissue

Next, we assessed whether euphol treatment could restore the morphological characteristics of inflamed colonic mucosa following DSS administration using scanning electron microscopy (SEM) [22]. SEM observations of the colonic mucosa in control healthy mice showed a normal epithelium with crypts and some granulated mast cells. The mucosal surface appeared to be subdivided by well-defined concave grooves and regular-shaped crypt openings containing mucin like material. A regular microvillus carpet makes the epithelial surface smooth and velvety (Fig. 2D). The DSS-group showed degenerated epithelium, severe inflammatory cell infiltration, widened grooves, dilatations of glandular crypts losing their regular shape by assuming fissure like aspects and depletion of goblet cells, leaving an irregular craterlike area (Fig. 2D). Relevantly, treatment with euphol (30 mg/kg, p.o.) following 7 days of DSS treatment significantly restored the architecture of the colon epithelium with a marked decrease in inflammatory cell infiltration compared with the DSS group (untreated mice). Similar to the control healthy group (non colitic), euphol treatment restored the microvillus carpet and made the epithelial surface smooth (Fig. 2D).

### Euphol inhibits cytokine local release and expression during experimental ulcerative colitis

**Figure 3:**
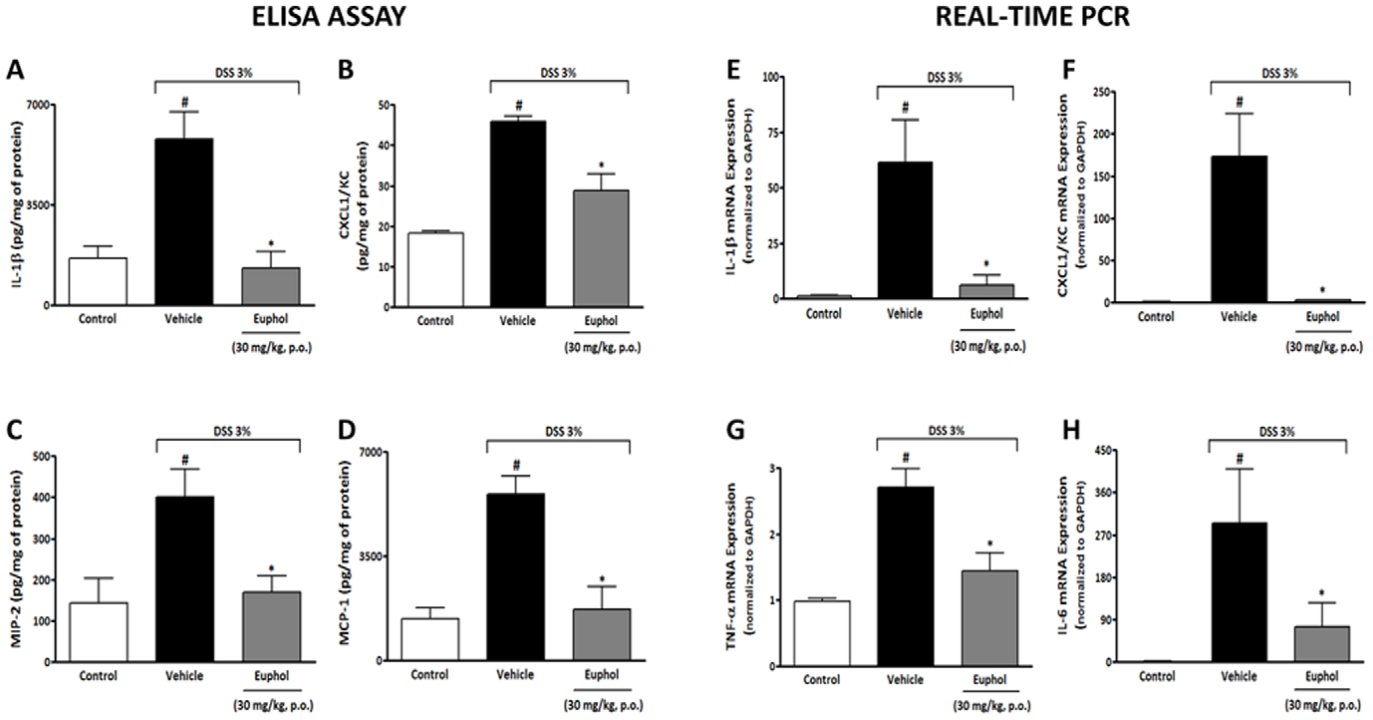
Preventive treatment with euphol changes colonic protein levels and mRNA expression of inflammatory mediators. At the end of 7 days, colon tissue was collected and processed for cytokine levels and mRNA expression. (A–D) Enzyme-linked immunosorbent assay. Preventive treatment with euphol (30 mg/kg, p.o.) reduced colonic levels of interleukin-1β (IL-1β) (A), keratinocyte-derived chemokine (CXCL1/KC) (B), macrophage inflammatory protein-2 (MIP-2) (C) and monocyte chemoattractant protein-1 (MCP-1) (D). (E–H) Real-time PCR. The same scheme of treatment with euphol also impaired the increase colonic mRNA expression of IL-1β (E), CXCL1/KC (F), tumor necrosis factor-α (TNF-α) (G) and interleukin-6 (IL-6) (H). The real-time PCR assay was performed in duplicate and GAPDH mRNA was used to normalize the relative amount of mRNA. Data are reported as means ± S.E.M. of 8 to 10 mice per group and is representative of three independent experiments. ^#^P<0.05 vs. control healthy group (non colitic); *P<0.05 vs. DSS-treated group.

It has been shown that selective blockade of IL-1R, CXCR2, CXCL1/KC, MCP-1, MIP-2, TNF-α and IL-6 significantly decreases severity of colitis and neutrophil/macrophage migration [4], [6], [23]. In this set of experiments, we investigated whether oral treatment with euphol could inhibit the levels and expression of pro-inflammatory cytokines/chemokine in the colonic tissues. Colonic levels of the pro-inflammatory cytokines IL-1β, CXCL1/KC, MIP-2 and MCP-1 were markedly elevated 7 days after the initiation of DSS treatment (Fig. 3 A–D). Preventive treatment with euphol (30 mg/kg, p.o.) significantly inhibited the levels of IL-1β by 80% (Fig. 3A), KC by 48% (Fig. 3B), MIP-2 by 65% (Fig. 3C) and MCP-1 by 80% (Fig. 3D) in colon tissue (P<0.05). In addition, DSS administration resulted in a pronounced increase in colonic IL-1β, CXCL1/KC, TNF-α and IL-6 mRNA expression (Fig. 3 E–H). Interestingly, euphol treatment (30 mg/kg), given orally during 7 day following DSS administration, significantly inhibited the up-regulated mRNA expression of IL-1β by 95% (Fig. 3E), CXCL1/KC by 100% (Fig. 3F), TNF-α by 40% (Fig. 3G) and IL-6 by 75% (Fig. 3H) (P<0.05). Such data suggest that the anti-inflammatory action of euphol is likely to be associated with its abilities to inhibit the release and expression of cytokines/chemokines as well as inhibit neutrophils influx into colonic tissue after DSS administration.

### Euphol reduces cytokine release from LPS-stimulated macrophages

**Figure 4:**
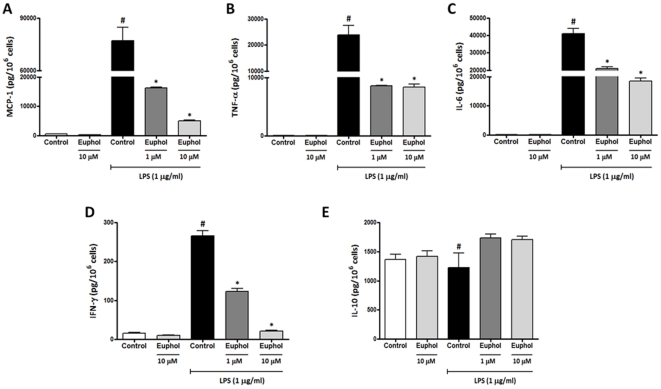
Euphol reduces pro-inflammatory cytokines and chemokines production in macrophages stimulates with lipopolysaccharide (LPS). Macrophage from bone marrow of naïve mice were stimulated with LPS (1 µg/ml) in the presence or absence of euphol (1 and 10 µM) for 24 hours, and the culture supernatants were analyzed for cytokine levels using cytokine bead array kit (CBA). Euphol incubation in dose-related manner reduced production of MCP-1 (A), TNF-α (B), IL-6 (C), IFN-γ (D), but increase the IL-10 levels (E). Data are reported as means ± SEM (n = 4) and is representative of two independent experiments. ^#^P<0.05 vs. control without LPS treatment (vehicle solution); *P<0.05 vs. LPS-treated group. Vehicle solution corresponds to 5% Tween 80 in medium.

The reduction in IL-1β, CXCL1/KC, MCP-1 and MIP-2 levels induced by euphol in DSS-treated colon could be just a consequence of decreased cellular adhesion or migration; inflammatory cells produce cytokines and chemokines, thus a decrease in the presence of these cells could also correspond to a decrease in their secreted cytokines/chemokines. To clarify whether euphol could also diminish the production of inflammatory mediators or modify the profile of cytokines produced by the inflammatory cells present in colonic tissue after inflammatory stimulus, we cultured primary bone marrow-derived macrophages and evaluated the influence of euphol on MCP-1, TNF-α, IL-6, IFN-γ and IL-10 production after *in vitro* stimulation with LPS (1 µg/ml, for 24 h), an important component of colitis-induced damage. Primary macrophages stimulated with LPS for 24 h increased MCP-1, TNF-α, IL-6 and IFN-γ levels (Fig. 4 A–E). *In vitro* pre-treatment (30 min) with euphol (1 and 10 µM) markedly blocked MCP-1, TNF-α, IL-6 and IFN-γ levels after LPS administration (Fig. 4). In addition, LPS administration decrease IL-10 levels and, interestingly, euphol (1 and 10 µM) increased IL-10 production in the macrophage culture after LPS administration (Fig. 4E).

### Euphol inhibits NOS2 and VEGF expression induced by DSS

**Figure 5:**
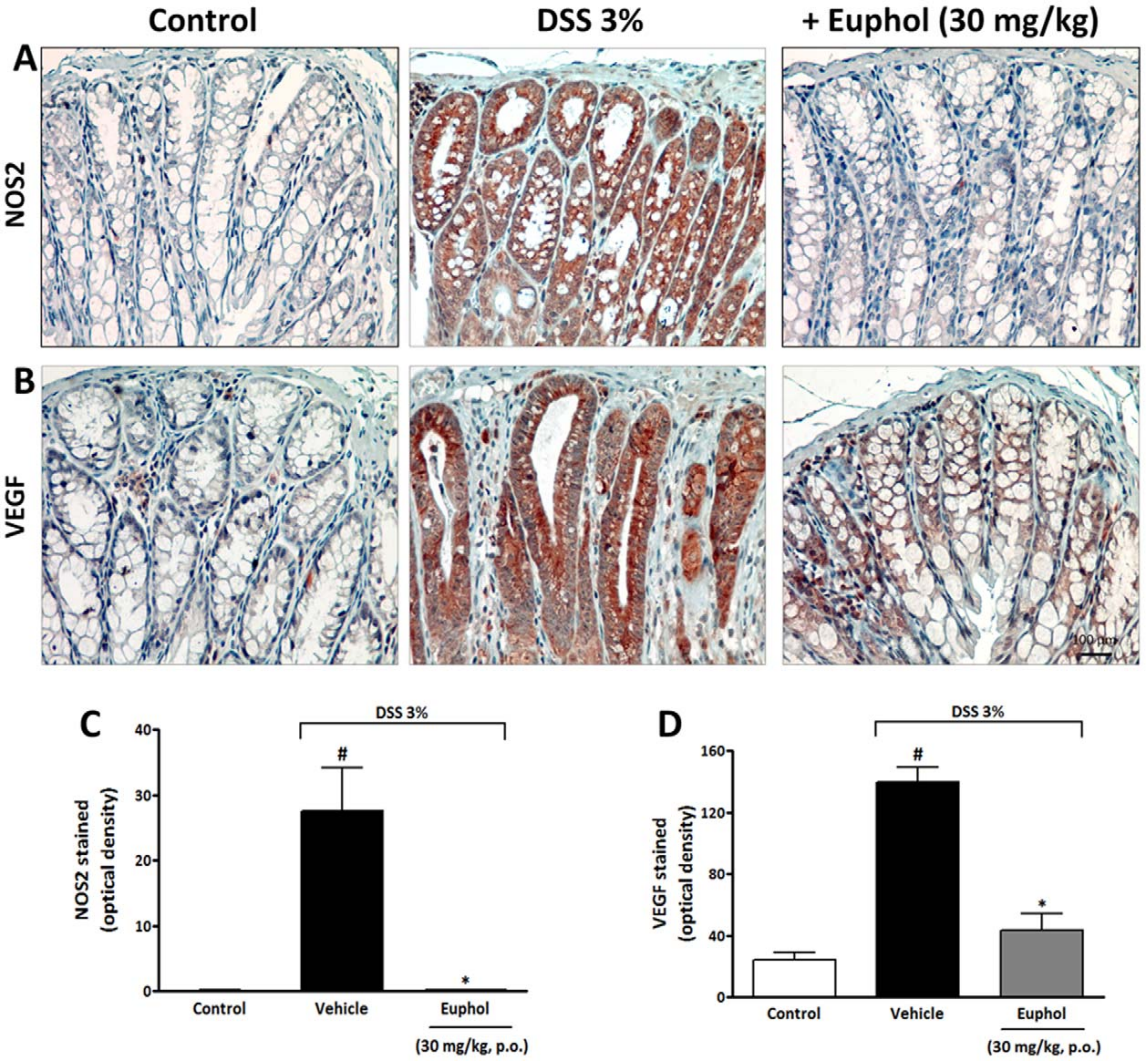
Euphol treatment inhibits NOS2 and VEGF expression in colonic tissue. After a 7-day euphol treatment, colon samples were processed for immunohistochemistry analysis. Preventive treatment with euphol (30 mg/kg, p.o.) significantly reduced NOS2 (A) and VEGF (B) immunostaining in the colon tissue after DSS-induced colitis in mice. Graphical representation of the immunostaining for NOS2 (C) and VEGF (D) expression evaluated in colon tissue. The mean intensity of NOS2 and VEGF staining were determined from image analysis and are represented as optical density. Scale bar corresponds to 100 µm and applies throughout. Each column represents the mean ± S.E.M. of 8 to 10 mice per group and is representative of three independent experiments. ^#^P<0.05 vs. control healthy group (non colitic); *P<0.05 vs. DSS-treated group.

Ulcerative colitis appears to be caused by a disruption of intestinal homeostasis and integrity, while up-regulated NOS2 expression in gut mucosa has been shown to cause apoptosis of epithelial cells [24]. Furthermore, it has been suggested that NOS2 is also involved in angiogenesis [25] a relevant phenomenon that has recently been demonstrated to be one of the major contributors to the pathogenesis of IBD [26]. Our present data corroborated with this observation by demonstrating that DSS-induced colitis increased NOS2 (Fig. 5 A,C) and VEGF expression (Fig. 5 B,D). Interestingly, preventive treatment with euphol (30 mg/kg) significantly blocked the increase in NOS2 and VEGF expression in colonic tissue (Fig. 5).

### Euphol inhibits inflammatory and enterocyte cells proliferation during inflammation bowel induced by DSS

**Figure 6:**
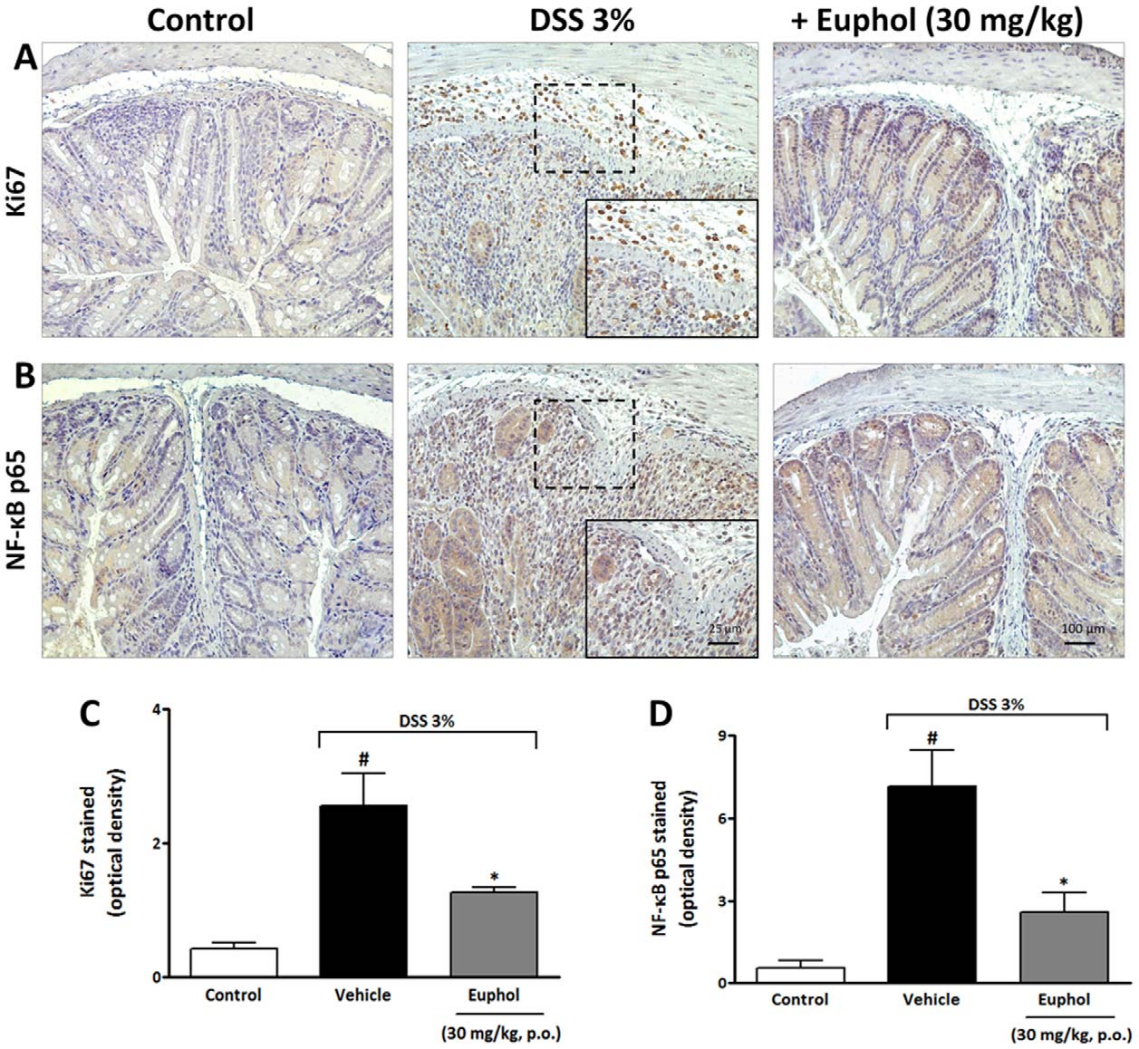
Euphol prevents inflammatory/enterocyte cells proliferation and NF-κB activation after DSS-induced colitis. Expression of Ki67 and phosphorylation of NF-κB was performed 7 days after administration of DSS (3%) or with vehicle in colonic tissues. Pre-treatment with euphol (30 mg/kg, p.o.), significantly inhibited proliferation index (Ki67) (A) and phosphorylation of p65 NF-κB (B) in colon tissue after DSS-induced colitis in mice. (A–B) Representative images of Ki67 and phospho-p65 NF-κB immunoreactivity in colon tissue. Scale bar corresponds to 100 and 25 mm (black square) respectively, and applies throughout. Graphical representation of the immunostaining for Ki67 (C) and phospho-p65 NF-κB (D) expression evaluated in colon tissue. The mean intensity of Ki67 and p65 NF-κB staining were determined from image analysis and are represented as optical density. Each column represents the mean ± S.E.M. of 8 to 10 mice per group and is representative of two independent experiments. ^#^P<0.05 vs. control healthy group (non colitic); *P<0.05 vs. DSS-treated group.

Ki-67 is a nuclear protein necessary for cell proliferation and is expected to play a central role in the inflammatory process [27]. To explore whether or not euphol could interfere with inflammatory and enterocyte cells proliferation we evaluated Ki-67 staining in colon tissue after DSS administration. Tissue sections from control mice exhibited very low levels of specific staining for Ki-67 in epithelial cells (Fig. 6). In contrast, at 7 days after DSS administration, the untreated (DSS) group showed intense immunostaining for Ki67 expression in colon tissue, mainly in the inflammatory cells, but also in enterocyte cells (Fig. 6 A,C). On the other hand, the preventive oral treatment with euphol (30 mg/kg, twice a day) during 7 days following DSS administration, significantly inhibited proliferation index in colon tissue, using monoclonal antibodies against ki67 (Fig. 6 A,C).

### Euphol prevents colonic NF-κB activation in the colon tissue after DSS administration

The transcriptional factor NF-κB is among the major pro-inflammatory signaling pathways involved in colitis [28], [29]. Herein, in order to further evaluate the possible mechanisms involved in the anti-inflammatory action of euphol, we next assessed whether the oral pre-treatment with euphol was able to decrease the activation of transcriptional factors NF-κB, in the colonic tissue after DSS administration. As shown in Fig. 6, low levels of NF-κB p65 was detected in the naive mouse colon; however, DSS-induced inflammation bowel, as expected, lead to a pronounced phosphorylation of p65 NF-κB in the colonic tissue after 7 days (Fig. 6 B,D). Interestingly, the pre-treatment with euphol (30 mg/kg, p.o.), significantly reduced the p65 NF-κB activation in the mouse colon tissue, thus strongly suggesting that inhibition of NF-κB activation seems to be the key mechanism through which this compound modulates intestinal inflammation (Fig. 6 B,D).

### Anti-inflammatory effect of euphol is mediated by inhibition of adhesion molecule expression in colonic tissue

**Figure 7:**
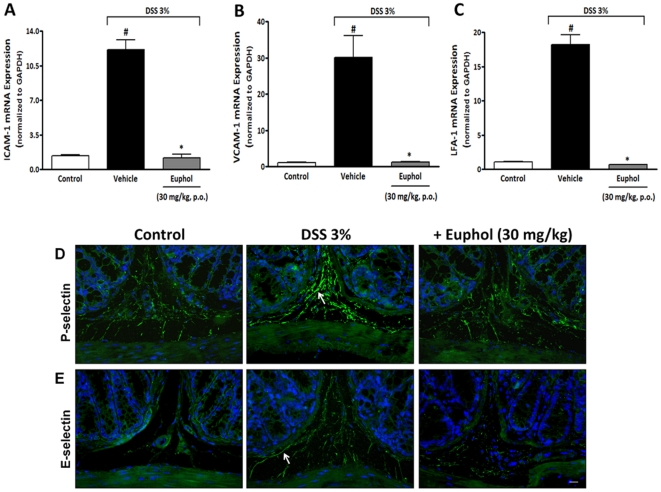
Preventive treatment with euphol blocks integrins and selectins expression in the colonic tissue after DSS-induced colitis. At the end of 7 days, colon tissue was collected and processed for mRNA expression and immunofluorescence. Preventive treatment with euphol (30 mg/kg, p.o.) reduced colonic mRNA expression of inter-cellular adhesion molecule 1 (ICAM-1) (A), vascular cell adhesion molecule-1 (VCAM-1) (B) and lymphocyte function-associated antigen 1 (LFA-1) (C). The real-time PCR assay was performed in duplicate and GAPDH mRNA was used to normalize the relative amount of mRNA. The same scheme of treatment with euphol also impaired the increase of P-selectin (D) and E-selectin (E). Representative images of P-selectin and E-selectin immunofluorescent stains were obtained on day 7 from control healthy mice, DSS-treated group and euphol (30 mg/kg, p.o.) treated group. Nuclei were stained with Hoechst (0.5 µl/ml). Scale bar corresponds to 50 µm and applies throughout. Data are reported as means ± S.E.M. of 8 to 10 mice per group and is representative of three independent experiments. ^#^P<0.05 vs. control healthy group (non colitic); *P<0.05 vs. DSS-treated group.

The process of leukocyte extravasations, a critical step in the inflammatory response, involves migration of leukocytes from the bloodstream toward colon tissue, which is orchestrated by the combined actions of cellular adhesion receptors (selectins and integrins) and chemotactic factors {Barreiro, #603}. In this set of experiments, we assessed whether oral treatment with euphol was able to decrease the expression of adhesion molecules in colonic tissue. In the first set of experiments, we assessed mRNA expression of adhesion molecules on day 7 after the induction of colitis. In the DSS group (untreated), a pronounced increase in colonic ICAM-1 (Fig. 7A), VCAM-1 (Fig. 7B) and LFA-1 (Fig. 7C) mRNA expression was observed. Interestingly, preventive treatment with euphol (30 mg/kg) markedly inhibited the up-regulated mRNA expression of ICAM-1, VCAM-1 and LFA-1 (Fig. 7) in colonic tissue. In addition, to determine the effect of euphol in regulating selectins, we performed immunofluorescence analysis of colon samples obtained from mice after DSS-induced colitis. Tissue sections from control healthy mice exhibited very low levels of specific staining for P-selectin (Fig. 7D) and E-selectin (Fig. 7E) in endothelial cells only. In contrast, 7 days after DSS administration, intense immunostaining for both P- and E-selectin expression in colon tissue was observed; however, preventive treatment with euphol (30 mg/kg, p.o.) during the 7 days after DSS administration resulted in a significant decrease in the positive immunostaining for P- and E-selectin (Fig. 7 D,E).

### Euphol treatment attenuates the severity of TNBS-induced colitis

**Figure 8:**
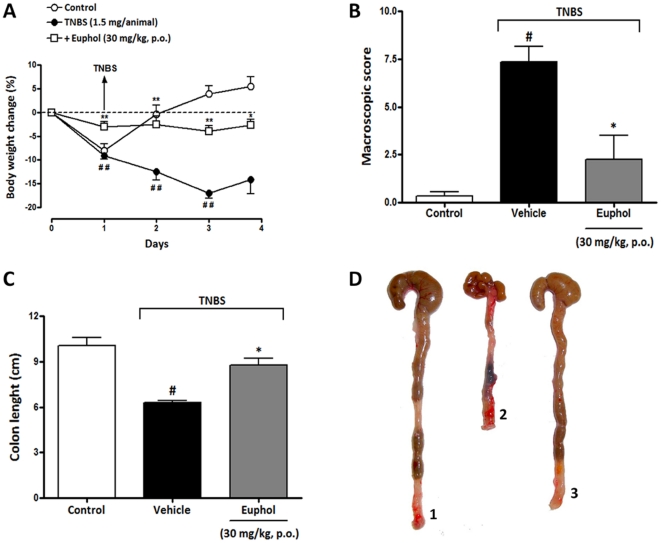
Therapeutic treatment with euphol protects mice against TNBS-induced acute colitis. Mice were given 100 µL of the TNBS (in 35% ethanol) and after 24 h, treated with euphol (30 mg/kg, p.o.). (A) The time-course of body weight changes on day 3 after TNBS-induced colitis. (B) Macroscopic score; (C) colon length after TNBS-induced colitis. (D) Representative photograph of colons from day 3 after the induction of TNBS-colitis. 1, Control healthy mice; 2, TNBS-treated (only vehicle administration); 3, TNBS plus euphol (30 mg/kg, p.o.). Each column represents the mean ± S.E.M. of 8 to 10 mice per group and is representative of two independent experiments. ^#^P<0.05 vs. control healthy group (non colitic); *P<0.05 vs. TNBS-treated group. Vehicle corresponds to 5% Tween 80 in saline 0.9% NaCl.

Recently, our group has shown that 72 h after TNBS administration, mice developed severe diarrhea, striking hyperemia, necrosis and inflammation accompanied by an extensive wasting disease, rectal prolapse and sustained weight loss [6]. To assess whether or not euphol treatment would also prove to be beneficial in another chemical model of colitis, we tested its effects on some parameters of colitis induced by the hapten TNBS, which constitutes a Crohn’s disease model. Rectal administration of TNBS dissolved in ethanol induced severe colitis in CD1 mice that was characterized by weight loss and diarrhea. Treatment with euphol (30 mg/kg, p.o.), twice a day, starting 24 h after TNBS administration (i.e., at a time point when colitis was already established), significantly reduced body weight loss (Fig. 8A). At this same dose, euphol also reduced the macroscopic damage score (Fig. 8B) and the reduction in colon length (Fig. 8 C,D).

## Discussion

Ulcerative colitis and Crohn’s disease are major forms of inflammatory bowel disease (IBD), a disease that affects millions of people worldwide and is characterized by chronic uncontrolled inflammation of intestinal mucosa [30], [31]. Despite intense interest, the pathogenesis of IBD still remains poorly understood; the imbalance between pro-inflammatory cytokines, such as TNF-α, IFN-γ, IL-1β, IL-6 and IL-12, and anti-inflammatory cytokines, such as IL-4, IL-10 and IL-11, is thought to play a pivotal role in modulating gut inflammation [32]. It has been reported that these inflammatory responses begin with a sudden infiltration of neutrophils and macrophages [33] with the activated macrophages producing a potent combination of broadly active inflammatory cytokines, including TNF- α, IL-1 and IL-6 [34]. Conventional therapy for IBD relies on the use of aminosalicylates, corticosteroids, immunosuppressive drugs and antibiotics [35]. Nowadays a new class of drugs has been used for the treatment of IBD, among them biologic agents such as anti-TNF therapy are noteworthy [36], [37]. However, these therapies for IBDs are usually associated with several side effects or clinical limitations for use. Therefore, there is a need for better therapeutic agents that effectively induce remission and/or alter the natural course of the disease with minimum or no side effects of treatment.

The use of medicinal plants or their active components is becoming an increasingly attractive approach for the treatment of various inflammatory disorders, such as rheumatoid arthritis [38], [39], multiple sclerosis {Ma, #622; Martin, #623; De Paula, 2008 #624} and ulcerative colitis [28]. Euphol, the alcohol derivate tetracyclic triterpene is the main constituent found in the sap of *Euphorbia tirucalli*, which is used as a folk therapy against syphilis, asthma, rheumatism, cancer and sarcoma [17]. In addition, a recent study have demonstrate that a biopolymeric fraction (BET) obtained from *E. tirucalli* showed dose-dependent anti-arthritic activity and demonstrated in vivo suppression of CD4^+^ and CD8^+^ T cells associated with the inhibition of intracellular IL-2 and IFN-γ, and inhibited vascular permeability and the migration of leucocytes at the site of the insult [21]. However, so far, there have been no reports about the effect of euphol on inflammatory bowel disease, such as UC and CD. Our results demonstrated the effectiveness of the isolated natural tetracyclic triterpene euphol in ameliorating two experimental model of colitis in mice. Euphol, given orally, consistently ameliorated the inflammatory symptoms associated with DSS-induced colitis, accompanied by a direct reduction in neutrophils migration, overproduction of pro-inflammatory mediators, macrophage infiltration and activation. Of interest, it was observed that euphol produced a marked decrease in adhesion molecule expression, such as integrins (ICAM-1, VCAM-1 and LFA-1) and selectins (P- and E-selectins), without compromising the integrity of the endothelial barrier, as seen by scanning electron microscopy. In addition, the present study clearly showed that euphol treatment consistently reduced activation of NOS-2, VEGF, Ki67 and p65 NF-κB. Furthermore, we also demonstrated that euphol treatment ameliorated TNBS-induced colitis, suggesting that the anti-inflammatory effect of euphol did not depend on the animal model of colitis used.

Several studies support a crucial role for neutrophils in mediating tissue injury and clinical symptoms in colitis [7], [8], [43]. Here, DSS-mediated colitis induces MPO activity and relevantly treatment with euphol prevented the increase in MPO activity. The reduction in neutrophils influx after euphol treatment observed in this study was associated with a decrease in colon damage. Interestingly, euphol was able to modulate the release and/or expression of pro-inflammatory cytokines/chemokines in colonic tissue. In this context, there is now a considerable amount of experimental evidence indicating that cytokines, such as MIP-2 and CXCL1/KC, play a pivotal role in the regulation of cell migration in colon tissue [6]. Taken together, these results suggest that euphol treatment contributes to a decrease in cell influx by diminishing the production of chemotactic factors, thus, in turn, ameliorating colon inflammation.

As discussed earlier, given that inflammatory cells up-regulated the production of cytokines/chemokines in pathological conditions, we next investigated whether the decreased levels and/or expression of colonic cytokines demonstrated by euphol treatment could be just a consequence of decreased cell migration. To further strengthen this view, we cultured primary bone marrow-derived macrophages and showed that euphol significantly reduced MCP-1, TNF-α, IL-6, and IFN-γ production in macrophages. Interestingly, *in vitro* pre-treatment (30 min) with euphol (1 and 10 µM) markedly increased IL-10 production, a relevant anti-inflammatory cytokine, in the macrophage culture after LPS administration. Considering these data, it is possible to suggest that euphol could modulate macrophage activation and in consequence decrease cytokine production, an effect which might contribute to the reduction of adhesion molecule expression.

Nitrosative stress caused by nitric oxide synthase 2 (NOS2 or iNOS)-derived nitric oxide (NO) is strongly associated with IBD progression and contributes to the pathogenesis of human IBD and experimental colitis [44]. Blocking NOS2 expression using gene knockout or specific inhibitors ameliorates the severity of experimental colitis [45], [46]. In addition, another relevant study has shown that NOS2 is implicated in the induction of VEGF [25], a cytokine that has a relevant role in angiogenesis and is increased in patients with IBD [26]. Additional evidence has indicated that local microvasculature and inflammation-dependent angiogenesis exert a relevant role in both human and murine IBD [26], [47], [48]. Here, we demonstrated that induction of colitis was associated with a significant increase in NOS2 and VEGF expression and the euphol treatment consistently blocked their up-regulation after DSS-induced colitis, suggesting that blocking intestinal inflammation and patho-angiogenesis through inhibiting NOS2 and VEGF expression during IBD progression may be, at least in part, the explanation of how euphol attenuates experimental colonic inflammation.

In addition, the pathway underlying the euphol protection of colon damage observed with preventive treatment could be associated with diminished of inflammatory and enterocyte cells proliferation (Ki67 immunostaining), which is a fundamental protein involved in cell proliferation [27]. This event seems to be associated with chronic inflammation and colon cancer and is increased under this condition [49]. In the present study, oral treatment with euphol notably reduced Ki67 protein activation in colonic tissue, suggesting that euphol ameliorates acute colitis, at least in parts, by reducing cell proliferation. However, these events might be an indirect consequence of the general improvement in inflammation mediated by euphol and need to be further clarified.

Nuclear factor κB is a critical transcription factor involved in a broad range of biological processes, including the regulation of immune and inflammatory responses which has been associated to the pathogenesis of colitis and other IBDs [50]. Activation of the NF-κB transcription factor induces the expression of several pro-inflammatory mediators, such as cytokines, chemokines and adhesion molecules, which in turn mediate the recruitment and activation of immune cells [51]. Herein, our results using the phospho-p65 NF-κB antibody demonstrated that euphol is able to inhibit the translocation of p65 into the nucleus, thus strongly suggesting that inhibition of NF-κB activation should be a key mechanism through which this natural tetracyclic triterpene modulates intestinal inflammation.

An increasing body of evidence has emerged indicating that cytokines and chemokines can up-regulate adhesion molecule expression [52], [53], [54], [55], [56]. The characteristic steps taken by leukocytes to extravasate from blood to the site of inflammation caused by either exogenous or endogenous stimuli have been recognized as the ‘three-step’ paradigm of inflammatory cell recruitment that involve rolling, activation and adhesion [16]. The interaction between leukocytes and the endothelium comprises a variety of adhesive and migratory molecular events, including low affinity transient and reversible rolling adhesion, integrin-dependent firm adhesive interaction and migratory events of leukocytes through the endothelium and beyond that, such as the penetration of the membrane and migration into the interstitial space [16]. The initial process of active leukocyte recruitment is the tethering or rolling of leukocytes, described as the initial selectin-mediated interaction between leukocytes and endothelial cells [57].

Multiple studies have indicated that antibody blockade of selectins inhibit leukocyte rolling *in vivo* [16], P-selectin^-/-^ mice show no leukocyte rolling *in vivo* [58] and double knockout mice for P- and E-selectin show a decrease in neutrophils mobilization [59]. The most important ligand for selectins is the P-selectin glycoprotein ligand-1 (PSGL-1), which is present on leukocytes and can bind to both P- and E-selectin [60]. Interestingly, PSGL-1 ligation in neutrophils by both P-selectin and E-selectin can result in activation of integrins, thus providing a link between rolling and the subsequent integrin-mediated firm adhesion [60]. Neutrophils and macrophages both use lymphocyte function-associated antigen 1 (LFA-1) (CD11a/CD18; αLβ2) and Mac-l (CD11b/CD18; αMβ2) for adhesion [61] in endothelial ICAMs, such as ICAM-1 and ICAM-2. Moreover, ICAM-1 expression is further increased after endothelial activation [62], and neutrophils-mediated adhesion to endothelial cells. In contrast, endothelial VCAM-1 is recognized by β1-integrin receptors predominantly found on lymphocytes and monocytes [63]. Another study demonstrated that induction of colitis in rats by TNBS is followed by up-regulation of endothelial VCAM-1, and suggests that VCAM-1 and constitutive ICAM-1 are major determinants of leukocyte recruitment to the inflamed intestine [64]. On the other hand, a relevant report established that VCAM-1 plays a central role in leukocyte recruitment in colitis and blockade of this adhesion molecule has higher therapeutic effect than immune neutralization of ICAM-1 or MAdCAM-1 in colitis experimental model. In the same study, chronic administration of anti-VCAM-1 antibody, but not anti-ICAM-1 or anti-MAdCAM-1, resulted in significant attenuation of colitis in terms of disease activity index, colon length, ratio of colon weight to length, and myeloperoxidase activity [65]. In this context, to gain further insight into the mechanisms through which euphol modulates cell migration and improves DSS-induced colitis in mice, we assessed the content of chemotactic factors and the expression of adhesion molecules after induction of colitis. Our results demonstrated that euphol almost totally suppressed the increase in mRNA expression of all integrins, accompanied by a significant decrease in the positive immunostaining of both selectin in colon tissue. Our present data suggest that euphol affects recruitment of leucocytes, especially neutrophils and macrophages, by regulating adhesion molecule expression in leucocytes and colonic endothelial cell. However, as described above the anti-inflammatory effect of euphol in the experimental colitis, could be justified mainly by the blockade of VCAM expression in the colonic tissue, however further studies are necessary to clarify this hypothesis.

In conclusion, our results demonstrate that orally-administered euphol has both preventive and therapeutic anti-inflammatory properties when assessed in two models of colitis in mice. The beneficial action of euphol in ameliorating colitis seems likely associated with its ability to prevent the expression of pro-inflammatory mediators and/or release in colonic tissue through inhibiting leukocyte influx (mainly neutrophils and macrophages) into inflammatory foci by blocking adhesion molecule expression, such as selectins (P- and E-selectins) and integrins (ICAM-1, VCAM-1 and LFA-1), without compromising the integrity of the endothelial barrier. Moreover, euphol treatment markedly inhibited the activation of NF-κB in the mouse colon tissue. These results suggest that euphol might constitute a potential candidate for the treatment of IBD.

## Materials and Methods

### Experimental animals

Male CD1 mice (8–10 weeks of age) were obtained from the Laboratório de Farmacologia Experimental (LAFEX), Universidade Federal de Santa Catarina (UFSC, Florianópolis, SC, Brazil) and housed in collective cages at 22 ± 1°C under a 12-h light/dark cycle (lights on at 07:00 h), with free access to food and tap water. All experiments were performed during the light phase of the cycle. The experimental procedures were previously approved by UFSC’s Committee on the Ethical Use of Animals and were carried out in accordance with Brazilian regulations on animal welfare (CEUA/UFSC protocol number 23080.030926/2010-62).

### DSS-induced colitis

This model of colitis was employed as previously described [66] and consisted of adding 3% w/v dextran sodium sulfate (30–50 kD, MP Biomedicals, Cleveland, OH, USA) to the animals’ drinking water for 5 days, followed by another 2 days during which they were offered DSS-free (i.e., plain) drinking water alone. Control healthy mice (non colitic) received plain drinking water at all times.

### TNBS-induced colitis

This colitis model employed here was originally described [67] and slightly modified [6]. After being deprived of food for 18–24 h with free access to a 5% glucose solution, mice were randomly divided into control healthy and colitis groups. Briefly, mice deprived of food for 1 day were lightly anaesthetized by administration of xylazine (10 mg/kg, i.p.) and ketamine (80 mg/kg, i.p.), and then a catheter (polyethylene PE-50) was carefully inserted into the colon (4 cm proximal to the anus). To induce colitis, TNBS (1.5 mg in 100 µL of 35% ethanol solution) was slowly administered (day 0). To assure the distribution of TNBS within the entire colon, mice were carefully maintained at a 45° angle (head down position) for 2 min and then returned to their cages. Four hours later, the animals were given free access to food and water. Throughout the experiments, mice were monitored for body weight loss and overall mortality. At 72 h following TNBS administration (day 3), i.e., 4 h after receiving the last injection, the animals were killed, and the colon was removed, dissected and opened lengthwise.

### Treatment protocols

The compound euphol was diluted in 5% Tween 80 solution made in saline (0.9% NaCl solution) and administered orally by gavage (p.o.). In the experiments involving DSS-induced colitis, mice received euphol (3, 10 and 30 mg/kg, p.o.) or vehicle (5% Tween 80 in saline 0.9% NaCl) twice a day from day 0 to day 7 (preventive treatment) or with 30 mg/kg from day 3 to day 7 (therapeutic treatment), and were sacrificed 4 h after receiving the last administration. After that the dose of 30 mg/kg (p.o.) euphol was used in subsequent experiments. To evaluate the potential therapeutic effect of euphol in TNBS-induced colitis, animals were orally treated by gavage with euphol (30 mg/kg, twice a day) or the corresponding vehicle (5% Tween 80 in saline 0.9% NaCl), starting 24 h after TNBS instillation (day 1) during 3 consecutive days (day 1–3). Vehicle solution (5% Tween 80 in 0.9% NaCl solution) was used in control experiments. The dose of each drug was chosen based on preliminary studies.

### Body weight change and disease activity index

Body weight was measured daily, starting on the day preceding DSS administration and up to 7 days thereafter in the DSS model. Body weight was also measured daily in the TNBS model, starting on the day preceding colitis induction (i.e., before fasting) and then again just prior to intracolonic TNBS or vehicle administration (after fasting) and up to 72 h after treatment. In the DSS model only, the clinical disease activity index (DAI) was measured daily using the protocol previously described [68], which ranged from 0 to 4 and was the sum of scores given for body weight loss (scored as: 0, none; 1, 1–5%; 2, 5–10%; 3, 10–20%; 4, over 20%), stool consistency (scored as: 0, well formed pellets; 2, loose stools; 4, diarrhea) and presence or absence of fecal blood (scored as: 0, negative hemoccult test; 2, positive hemoccult test; 4, gross bleeding).

### Macroscopic and microscopic colonic damage

In the DSS model at the end of the 7-day period, colons were removed and examined for: weight, the consistency of the stool found within as well as gross macroscopic appearance and length, which was measured from 1 cm above the anus to the top of the cecum. Macroscopic damage was assessed according previously [69], as the sum of scores attributed to stool condition (0, normal well-formed fecal pellets; 1, loosely shaped moist pellets; 2, amorphous, moist, sticky pellets; 3, diarrhea; plus 1 for presence of blood in stool), colon damage (0, no inflammation; 1, reddening, mild inflammation; 2, moderate or more widely distributed inflammation; 3, severe and/or extensively distributed inflammation), colon weight loss (0, for <5%; 1, for 5–14%; 2, for 15–24%; 3, for 25–35%; 4, for >35%) and colon length shortening (0, for <5%; 1, for 5–14%; 2, for 15–24%; 3, for 25–35%; 4, for >35%), with up to a maximum total score of 15. To evaluate microscopic colon damage by light microscopy, samples of the distal colon were fixed immediately in 10% formaldehyde solution, embedded in paraffin, cut into 5 µm thick transversal sections, mounted on glass slides, deparaffinized and stained with hematoxylin and eosin stain (H&E). In each specimen, six random fields of view were analyzed by two double-blind observers, using Sight DS-5M-L1 digital camera connected to an Eclipse 50i light microscope (both from Nikon, Melville, NY, USA). The intensity of microscopic colonic damage was assessed according to the scoring system described by Rath et al. [70] and modified by Van der Sluis et al. [71].

In the TNBS model, at 3 days after TNBS administration, mice were sacrificed and their colons removed and rinsed with saline, and macroscopic colonic damage was evaluated using the following scoring system: 0, no damage; 1, hyperemia without ulcers; 2, hyperemia with bowel wall thickening but no ulcers; 3, one site of ulceration without bowel wall thickening; 4, two or more sites of ulceration or inflammation; 5, 0.5 cm of inflammation and major damage; 6, at least 1 cm of major damage (for every additional 0.5 cm of damage, the score was increased by one to a maximum of 10); plus 1 for presence of diarrhea or stricture; plus 1 or 2 for presence of mild or severe adhesions, respectively [72].

### Scanning electron microscopy (SEM)

SEM was used to visualize topographical alterations of the luminal surface of the colon from control healthy, untreated and treated mice. Fragments of 2 mm thickness from the mid-colon were pre-fixed in the field in 2.5% glutaraldehyde in 0.1M sodium phosphate buffer (pH 7.4) and kept on ice. The specimens were then rinsed in the same buffer, post-fixed in 2% osmium tetroxide (OsO4) for 4 h, dehydrated in a graded ethanol series (30% to 100%) and dried in hexamethyldisilazane (HMDS). The samples were glued onto stubs, covered with gold particles (Bio-Rad SC502, Hertfordshire, UK) and observed under a scanning electron microscope (JEOL JSM-6390LV, Tokyo, Japan).

### Myeloperoxidase assay

Neutrophil infiltration into the colon was assessed indirectly by measuring myeloperoxidase (MPO) activity. Mid-colon segments were homogenized in ethylenediaminetetraacetic acid (EDTA)/NaCl buffer (pH 4.7) and centrifuged at 10,000 rpm for 15 min at 4°C. The pellet was ressuspended in 0.5% hexadecyltrimethyl ammonium bromide buffer (pH 5.4) and frozen in liquid nitrogen and thawed repeatedly three times. Samples were then centrifuged again (10,000 rpm, 15 min, 4°C) and 25 µl of the supernatant was used for the MPO assay. The enzymatic reaction was assessed by the addition of 25 µl of 1.6 mM tetramethylbenzidine (TMB) in 80 mM NaPO4, plus 100 µl of 0.3 mM H_2_O_2_. MPO activity was measured spectrophotometrically at 650 nm and the results are expressed as optical density (OD) per milligram of tissue.

### Determination of cytokine levels

Mid-colon segments were homogenized in phosphate buffer containing 0.05% Tween 20, 0.1 mM phenylmethylsulfonyl fluoride, 0.1 mM benzethonium chloride, 10 mM EDTA and 20 IU aprotinin A. The homogenates were centrifuged at 3,000×g for 10 min and the supernatants stored at −80°C until assays for the determination of levels of the cytokines IL-1β, keratinocyte-derived chemokine (CXCL1/KC), monocyte chemoattractant protein-1 (MCP-1) and macrophage-inflammatory protein-2 (MIP-2) were carried out. The amount of protein in each sample was measured using the Bradford method [73], using bovine serum albumin as a standard. The levels of each cytokine were evaluated using enzyme-linked immunosorbent assay (ELISA) kits according to the manufacturer’s recommendations (R&D systems, Minneapolis, MN, USA) and the results are expressed in pg/mg of protein in each sample.

### Real-time quantitative PCR

Total RNA was extracted from mid-colon samples taken from anesthetized mice treated with DSS using the Trizol protocol (Invitrogen, Carlsbad, CA, USA) and its concentration was determined by NanoDrop™ 1100 (NanoDrop Technologies, Wilmington, DE, USA). A reverse transcription assay was performed as described in the M-MLV Reverse Transcriptase protocol according to the manufacturer’s instructions. cDNA (300 ng) was amplified in triplicate using TaqMan® Universal PCR Master Mix Kit with specific TaqMan Gene Expression target genes, the 3’ quencher MGB and FAM-labeled probes for mouse TNF-α (Mm00443258_m1), IL-1β (Mm01336189_m1), CXCL1/KC (Mm00433859_m1), IL-6 (Mm99999064_m1), ICAM-1 (Mm005616024_g1), VCAM-1 (Mm01320970_m1), lymphocyte function- associated antigen 1 (LFA-1, Mm01278854_m1) and GAPDH (NM_008084.2) (which was used as an endogenous control for normalization). The PCR reactions were performed in a 96-well Optical Reaction Plate (Applied Biosystems, Foster City, CA, USA). The thermocycler parameters were as follows: 50°C for 2 min, 95°C for 10 min, 50 cycles of 95°C for 15 sec and 60°C for 1 min. Expression of the target genes was calibrated against conditions found in control non-colitic animals, i.e., those that received vehicle (5% Tween 80 in saline 0.9% NaCl).

### Tissue and immunostaining

Seven days after DSS-induced experimental colitis, animals were sacrificed and each portion of the distal colon was fixed immediately in 4% paraformaldehyde in 0.1 M phosphate buffer (pH 7.4). Colons were removed and post-fixed 24 h in the same solution, following embedding in paraffin and sections (5 µm) mounted on glass slide and deparaffinized. Immunohistochemical analysis was performed on paraffin-embedded colon tissue (5 µm) using polyclonal rabbit anti-NOS2 (1∶300), monoclonal mouse anti-VEGF (1∶300), polyclonal rabbit anti-Ki-67 (1∶300) and monoclonal mouse anti-phospho-p65 NFκB (1∶50) according to the method described previously [74]. After quenching of endogenous peroxidase with 1.5% hydrogen peroxide in methanol (v/v) for 20 min, high-temperature antigen retrieval was performed by immersion of the slides in a water bath at 95 to 98°C in 10 mmol/L trisodium citrate buffer, pH 6.0, for 45 min. The slides were then processed using the VECTASTAIN® Elite ABC reagent (Vector Laboratories, Burlingame, CA), according to the manufacturer’s instructions. After the appropriate biotinylated secondary antibody, sections were developed with 3,3'-diaminobenzidine (Dako Cytomation, Glostrup, Denmark) in chromogen solution for the exact same amount of time and counterstained with Harris’s hematoxylin. For immunofluorescence, sections 5 µm in thickness were deparaffinized and blocked with 2% BSA in 0.3% Triton X-100 for 1 h at room temperature and incubated overnight at 4°C with antibodies specific for P-selectin (polyclonal goat; 1∶200 dilution) and E-selectin (polyclonal goat; 1∶100 dilution), followed by fluorescein isothiocyanate-conjugated donkey goat-specific secondary antibody (1∶50 dilution; Vector Laboratories, Burlingame, CA) for 2 h at room temperature. Nuclei were stained with Hoechst (0.5 µl/ml; Molecular Probes). Images were acquired using a Sight DS-5M-L1 digital camera connected to Eclipse 50i light microscope fluorescence (both from Nikon, Melville, NY, USA). Four ocular fields per section (5–10 mice per group) were captured and a threshold optical density that best discriminated staining from the background was obtained using the NIH ImageJ 1.36b imaging software (NIH, Bethesda, MD, USA). For immunohistochemistry analysis, total pixel intensity was determined and data are expressed as optical density (OD). For immunofluorescence, signal intensities were collected in triplicate and microscopy data were acquired by two investigators ‘blinded’ to the identity of the disease group.

### Murine bone marrow-derived macrophages

CD1 mice were sacrificed by cervical dislocation. Total bone marrow was obtained from mice by flushing the femurs and tibiae with Dulbecco modified eagle medium (DMEM), as previously described [75]. Briefly, bone marrow mononuclear phagocyte precursor cells were propagated in suspension by culturing in macrophage medium (DMEM containing glucose, supplemented with 2 mM L-glutamine, 10% FCS, 10 mM Hepes, 100 µg/ml streptomycin, 100 U/ml penicillin (all from Sigma-Aldrich) supplemented with 20% L929 cell-conditioned medium (as a source of M-CSF). Cells were incubated at 37°C in 5% CO_2_ air and fed on day 5 by replacing the medium supplemented with 20% L929 cell-conditioned medium. Cells were harvested on day 7, and 1×10^6^ cells/ml were cultured in a 96-well cell culture plate for 24 h. Afterwards, adherent cells were stimulated for 24 h with LPS (1 µg/ml) in the presence or absence of euphol (1 and 10 µM) in a final volume of 250 µl/well. Control group corresponds to 5% Tween 80 in medium with/without LPS treatment. After stimulation, the plate was centrifuged (200×g/10 min) and the cell-free supernatant was collected and stored at −70°C for cytokine determination. A cytokine bead array Mouse Kit Inflammation was used to measure MCP-1, TNF-α, IL-6, IFN-γ and IL-10 secretion in the supernatant. The data were acquired using BD FACSCanto II (BD Biosciences, San Diego, CA, USA) and analyzed using FCAP Array software (BD Biosciences, San Diego, CA, USA).

### Drugs and reagents

The sap from *Euphorbia tirucalli* was initially extracted with hexane and the resulting precipitate was extracted with n-butanol. The most lipophilic compounds present in the methanol fraction were purified by means of high performance liquid chromatography (HPLC) analysis. Further purification of the compounds was carried using Sephadex G-75 in a mixture of hexane-ethyl acetate. The euphol chemical characterization was conducted by means of nuclear magnetic resonance (NMR) and mass spectroscopic data. The tetracyclic triterpene euphol used in this study showed a purity >95%. Dexamethasone, H_2_O_2_, Tween 20, Tween 80, EDTA, aprotinin, phosphate buffered saline (PBS), H&E, 3,3,5,5-tetramethylbenzidine, H_2_O_2_ and TNBS were purchased from Sigma Chemical Co. (St. Louis, MO, USA). Formaldehyde was obtained from Merck (Frankfurt, Darmstadt, Germany). Anti mouse-KC and the DuoSet kits for IL-1β/IL-1F2, MCP-1 and MIP-2 were obtained from R&D Systems (Minneapolis, MN, USA). Bradford reagent was purchased from Bio-Rad Laboratories (Richmond, CA, USA). Polyclonal rabbit anti-NOS2 was purchased from Thermo Fisher Scientific Inc. (Fremont, CA, USA). Monoclonal mouse anti-VEGF was purchased from Santa Cruz Biotechnology (Santa Cruz, CA, USA). Monoclonal mouse anti-phospho-p65 NF-κB was purchased from Cell Signaling Technology (Danvers, MA, USA) and polyclonal rabbit anti-Ki-67 from purchased Abcam (Cambridge, MA, USA). Secondary antibody Envision Plus, streptavidin–HRP reagent, and 3,3-diaminobenzidine chromogen were purchased from DakoCytomation (Carpinteria, CA, USA). Trizol and M-MLV reverse transcriptase were purchased from Invitrogen (Carlsbad, CA, USA). Primers and probes for mouse TNF-α (Mm00443258_m1), IL-1β (Mm01336189_m1), CXCL1/KC (Mm00433859_m1), IL-6 (Mm99999064_m1), ICAM-1 (Mm005616024_g1), VCAM-1 (Mm01320970_m1), LFA-1 (Mm01278854_m1), GAPDH (NM_008084.2) and TaqMan® Universal PCR Master Mix Kit were purchased from Applied Biosystems (Foster City, CA, USA). The CBA Mouse Inflammation Kit was purchased from BD Biosciences (San Diego, CA, USA). The compound euphol (3, 10 and 30 mg/kg) was diluted in a 5% Tween 80 solution made in saline (0.9% NaCl solution) and administered orally by gavage (p.o.). All other drugs were made in physiological saline (0.9% NaCl solution).

### Statistical analysis

All data were expressed as means ± S.E.M. (n = 8−10 animals/group). Statistical analysis was performed using Kruskal-Wallis followed by Dunn’s test for non-parametric data, one-way ANOVA followed by Newman-Keuls test for parametric data. All analysis was conducted using GraphPad Prism 4 software (GraphPad Software Inc., San Diego, CA, USA). Differences with *p*≤0.05 were considered to be statistically significant.
